# Effects of Cold-inducible RNA-binding Protein (CIRP) on Liver Glycolysis during Acute Cold Exposure in C57BL/6 Mice

**DOI:** 10.3390/ijms20061470

**Published:** 2019-03-23

**Authors:** Peng Liu, Ruizhi Yao, Hongzhao Shi, Yang Liu, Shuai Lian, Yuying Yang, Huanmin Yang, Shize Li

**Affiliations:** College of Animal Science and Veterinary Medicine, Heilongjiang Bayi Agricultural University, Daqing 163319, China; 18346663533@163.com (P.L.); 15848955365@163.com (R.Y.); 15776557846@163.com (H.S.); lymissive@163.com (Y.L.); lianlianshuai@163.com (S.L.); yalele258@sina.com (Y.Y.); byndyhm@163.com (H.Y.)

**Keywords:** CIRP, cold exposure, liver, glucose metabolism, apoptosis

## Abstract

Cold-inducible RNA-binding protein (CIRP) is a stress-responsive protein involved in several signal transduction pathways required for cellular function, which are associated with apoptosis and proliferation. The present study aimed to investigate the possible effects of CIRP-mediated regulation of glucose metabolism in the liver following acute cold exposure. The livers and serum of male C57BL/6 mice were collected following cold exposure at 4 °C for 0 h, 2 h, 4 h, and 6 h. Glucose metabolic markers and the expression of glucose metabolic-related proteins were detected in the liver. Acute cold exposure was found to increase the consumption of glycogen in the liver. Fructose-1,6-diphosphate (FDP) and pyruvic acid (PA) were found to show a brief increase followed by a sharp decrease during cold exposure. Anti-apoptotic protein (Bcl-2) expression was upregulated. CIRP protein expression displayed a sequential increase with prolonged acute cold exposure time. Acute cold exposure also increased the level of protein kinase B (AKT) phosphorylation, and activated the AKT-signaling pathway. Taken together, these findings indicate that acute cold exposure increased the expression of CIRP protein, which regulates mouse hepatic glucose metabolism and maintains hepatocyte energy balance through the AKT signaling pathway, thereby slowing the liver cell apoptosis caused by cold exposure.

## 1. Introduction

Any organisms that live at a particular environmental temperature are affected by changes in temperature. When organisms are exposed to an excessively cold environment, it often causes tissue histopathological damages [1]. Cold exposure was reported to induce liver oxidative damage and metabolic changes as well [2]. Cold also provokes a series of hypothermic cellular effects, including reduced oxygen consumption, decreased metabolic rate, and changes in the state of redox and gene expression [3]. This results in metabolic disorders of the organism’s body, synthesis of total protein is suppressed, immune function is reduced, and damage is caused to various organs of the animal body; if serious enough, such issues can be life-threatening.

Acute cold exposure significantly increases systemic energy expenditure and causes substantial metabolic changes, including increased ingestion, decreased insulinemia, increased hepatic glucose production, and increased glucose and fat utilization in peripheral tissues [4]. It has been reported that liver glycogen exposed to cold decreases rapidly, and the key enzymes required for the synthesis of glycogen in the liver are up-regulated, which eventually leads to the consumption of ATP and apoptosis in cells [5,6]. Although energy metabolism is the basis for organism survival, breeding, and other important life activities, it is also closely related to human obesity, type II diabetes, and other metabolic syndromes. Therefore, studying the regulation of energy metabolism has great significance for improving human health. 

Cold-induced RNA binding protein (CIRP) was the first cold shock protein identified in mammals in 1997. CIRP was found to increase in a dose-dependent manner in response to UV radiation or UV mimetics [7], and was subsequently further identified as a protein induced under conditions of mild cold shock [8]. Recently, increasing research shows that CIRP is associated with a variety of physiological and pathological processes, including the regulation of hibernating activities in amphibians [9,10], the growth and development of humans and animals (e.g., neurodevelopment, embryonic development, and reproductive development) [11,12,13,14,15,16], expression of circadian rhythm genes [17,18], carcinogenesis [19,20,21], and immune response [22,23,24]. In addition, CIRP also plays a crucial role in the cryoprotection of tissues and organs, as well as treating brain injury [25,26,27]. Despite a large number of studies, it remains unclear whether CIRP can exert metabolic regulation under low temperatures to protect tissues from cryogenic threats. In the present study, we report that as a low-temperature response protein, CIRP may participate in the control of low-temperature metabolism in the organism, and we explore the potential mechanisms for this.

## 2. Results

### 2.1. Acute Cold Exposure Induced Changes in Core Body Temperature

**Figure 1:**
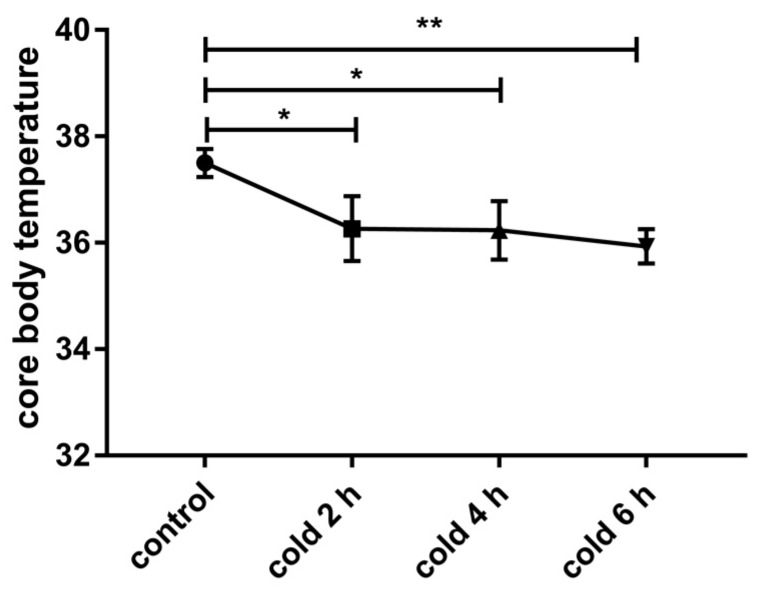
Effect of acute cold exposure on core body temperature in C57BL/6 mouse. The data are presented as the mean ± SEM (*n* = 8). Statistically significant differences are indicated; * *p* < 0.05 and ** *p* < 0.01.

Our results show that acute cold exposure induces a decrease in core body temperature in mice (Figure 1; *p* < 0.05 and *p* < 0.01), and core body temperature drops to 35.9 °C at cold exposure at 6 h.

### 2.2. Acute Cold Exposure Induces A Plasma Glucose, Insulin, and Glucagon Time-Course

**Figure 2:**
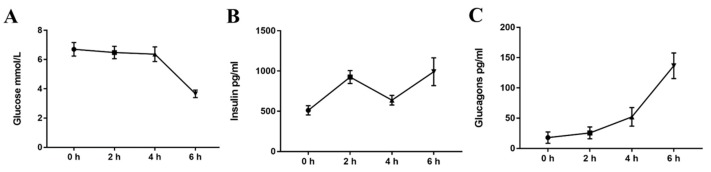
Effect of acute cold exposure on the level of (**A**) glucose, (**B**) insulin, and (**C**) glucagon in C57BL/6 mouse plasma. The data are presented as the mean ± standard error of the mean (SEM) (*n* = 8). Statistically significant differences are indicated; * *p* < 0.05 and ** *p* < 0.01.

To identify the effect of cold exposure on metabolism in mice, we assessed the level of plasma glucose, insulin, and glucagon (GC) in the serum of mice in both the control and cold-exposed groups. As shown in Figure 2, compared with the normal temperature control group, while the level of blood glucose was significantly decreased in the cold exposure group at 6 h, there was no significant change in the 2 h and 4 h cold exposure groups (*p* < 0.01). The level of glucagon increased in the cold-exposed groups, with significant differences at 4 h and 6 h (*p* < 0.01). However, insulin levels fluctuated in the cold-exposed groups. 

### 2.3. Acute Cold Exposure-Induced Changes in Hepatic Fructose-1,6-diphosphate (FDP) and Pyruvic Acid (PA) in Mice

**Figure 3:**
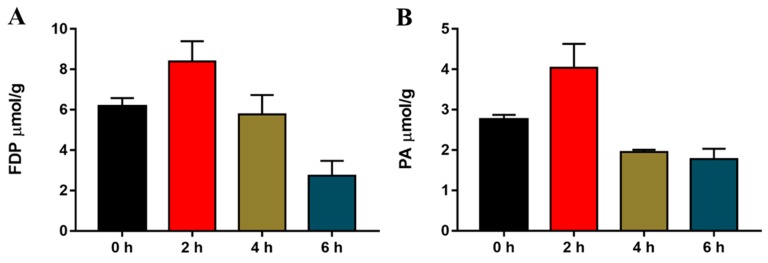
Effect of acute cold exposure on the level of glycolytic intermediates: (**A**) fructose-1,6-diphosphate (FDP) and (**B**) pyruvic acid (PA) in C57BL/6 mouse livers. The data are presented as the mean ± SEM (*n* = 8). Statistically significant differences are indicated; * *p* < 0.05 and ** *p* < 0.01.

To determine the effect of acute cold exposure on liver glycolysis in mice, we determined the glycolytic intermediates of FDP and PA. The mouse FDP and PA content before and after cold exposure were measured. As shown in Figure 3, the level of both FDP and PA in the livers of mice exhibited significant changes during cold exposure. Compared with the normal temperature control group, the FDP and PA content were significantly elevated in the 2 h cold exposure group, which decreased to levels comparable to the control group in the 4 h cold-exposed group, and continued to decrease further in the 6 h cold-exposed group.

### 2.4. Acute Cold Exposure Induces Glycogen Depletion 

**Figure 4:**
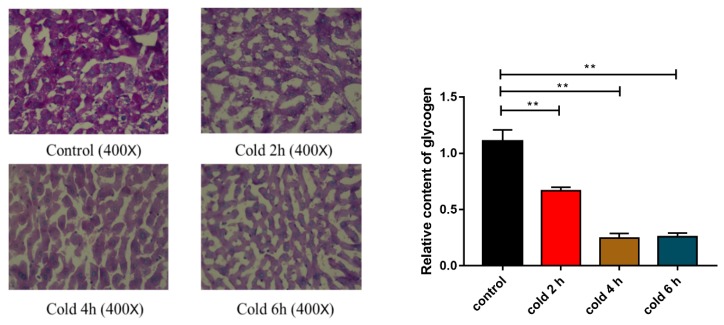
Effect of acute cold exposure on glycogen content in C57BL/6 mouse livers. The data are presented as the mean ± SEM (*n* = 8). Statistically significant differences are indicated; * *p* < 0.05 and ** *p* < 0.01.

In Periodic Acid-Schiff (PAS) staining, the glycogen was stained purple and the nuclei of the hepatic cells were stained blue. The depth of purple is proportional to the glycogen content. As shown in Figure 4 (*p* < 0.01), compared with the control group, there was a significant reduction in the glycogen content in the livers of each of the groups of mice.

### 2.5. Acute Cold Exposure Upregulates CIRP Expression

**Figure 5:**
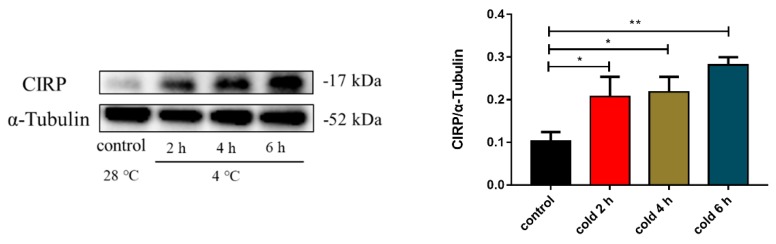
Effect of acute cold exposure on the level of cold-inducible RNA-binding protein (CIRP) expression in C57BL/6 mouse livers. The data are presented as the mean ± SEM (*n* = 8). Statistically significant differences are indicated; * *p* < 0.05 and ** *p* < 0.01.

Previous studies have shown that cold exposure can lead to the upregulation of CIRP protein expression. The results of this study showed that CIRP protein expression displayed sequential changes with prolonged acute cold exposure time, and there was a significant difference at 6 h compared with the control group (Figure 5; *p* < 0.05 and *p* < 0.01). Thus, we further validated that acute cold exposure can affect the level of CIRP protein expression. 

### 2.6. Acute Cold Exposure Activates the AKT Signaling Pathway

**Figure 6:**
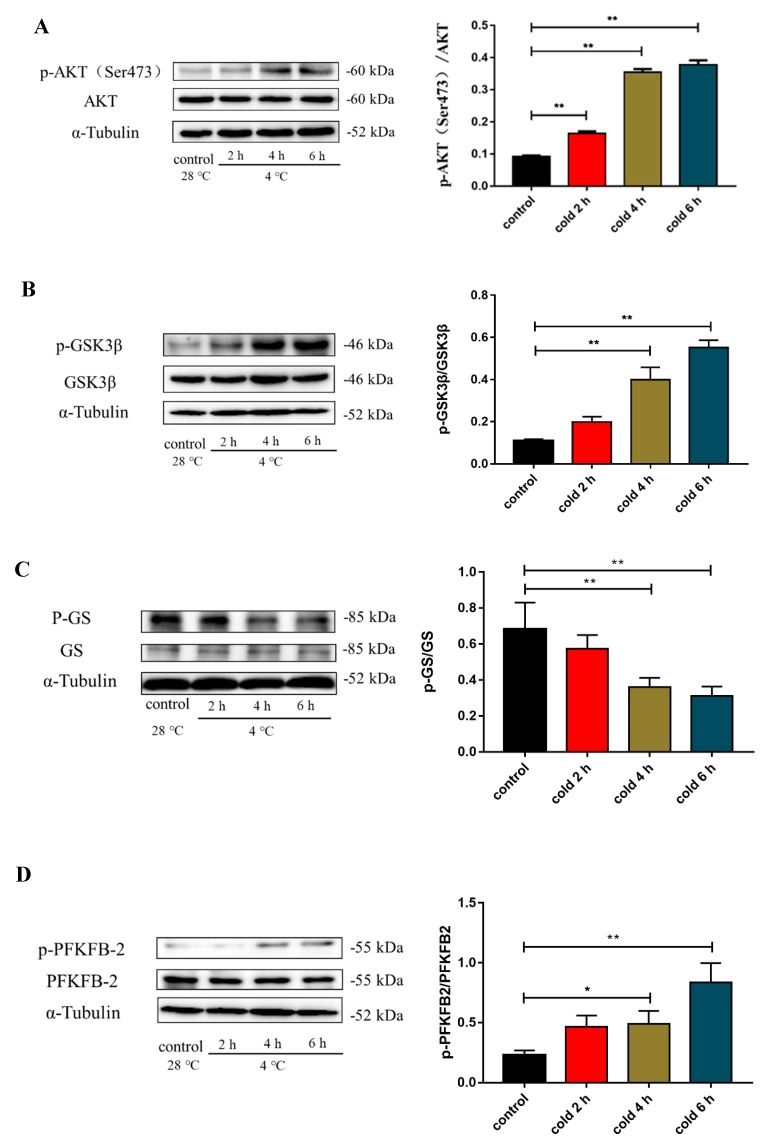
Effect of acute cold exposure on glucose metabolism in C57BL/6 mouse livers. (**A**) The level of protein kinase B (AKT) protein and phosphorylation status (Thr308) in the liver; (**B**) the level of glycogen synthase kinase-3β (GSK3β) protein and phosphorylation status (Ser9) in the liver; (**C**) the level of glycogen synthase (GS) protein and phosphorylation status (Ser641) in the liver; (**D**) the level of 6-phosphofructo-2-kinase/fructose-2,6-biphosphatase 2 (PFKFB2) protein and phosphorylation status (Ser483) in the liver. The data are presented as the mean ± SEM (*n* = 8). Statistically significant differences are indicated; * *p* < 0.05 and ** *p* < 0.01.

To investigate the mechanism of glucose metabolism under cold exposure, we measured the AKT signaling pathway by measuring the level of liver AKT, glycogen synthase kinase-3β (GSK3β), and 6-phosphofructo-2-kinase/fructose-2,6-biphosphatase 2 (PFKFB2) protein expression and phosphorylation. As shown in Figure 6, cold exposure enhanced the level of p-AKT (Figure 6A; *p* < 0.01), p-GSK3β (Figure 6B; *p* < 0.01), and p-PFKFB2 (Figure 6D; *p* < 0.01) protein expression, and reduced p-GS (Figure 6C; *p* < 0.05 and *p* < 0.01) protein expression.

### 2.7. Acute Cold Exposure Induces Apoptosis

**Figure 7:**
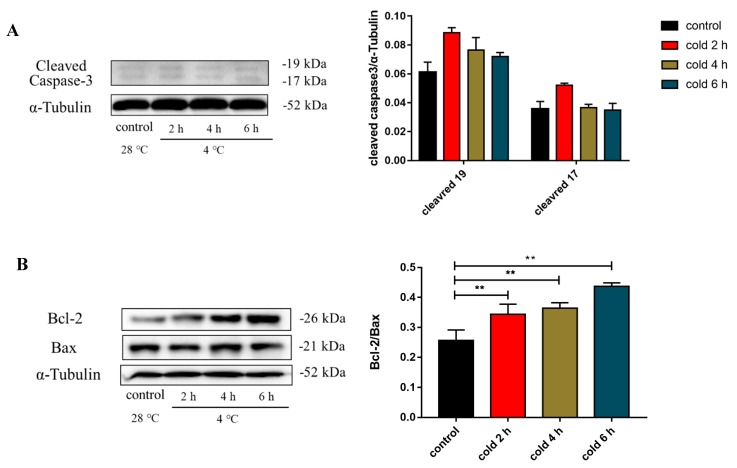
Effect of acute cold exposure on the expression of (**A**) caspase-3, (**B**) Bcl-2, and Bax in the livers of C57BL/6 mice. The data are presented as mean ± SEM (*n* = 8). Statistically significant differences are indicated; * *p* < 0.05 and ** *p* < 0.01.

To determine the influence of cold exposure on cell apoptosis, the expression of caspase 3, Bcl-2, and Bax in the liver was measured. As shown in Figure 7A, no significant change in the expression of cleaved caspase 3 was observed between the experimental and control groups. The ratio of Bcl-2/Bax protein expression (Figure 7B; *p* < 0.01) in the livers of the experimental mouse groups was significantly elevated compared to that of the control group.

### 2.8. Effect of AKT on Glucose Metabolism in C57BL/6 Mouse Liver under Cold Exposure

**Figure 8:**
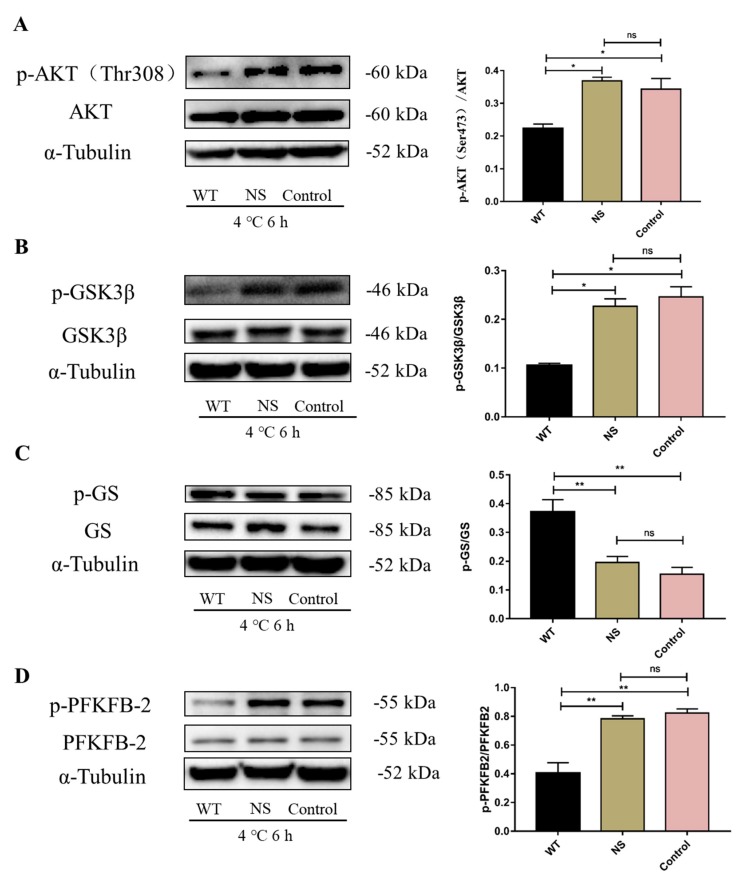
Effect of AKT on glucose metabolism in C57BL/6 mouse livers under cold exposure. (**A**) The level of protein kinase B (AKT) protein and phosphorylation status (Thr308) in the liver; (**B**) the level of glycogen synthase kinase-3β (GSK3β) protein and phosphorylation status (Ser9) in the liver; (**C**) the level of glycogen synthase (GS) protein and phosphorylation status (Ser641) in the liver; (**D**) the level of 6-phosphofructo-2-kinase/fructose-2,6-biphosphatase 2 (PFKFB2) protein and phosphorylation status (Ser483) in the liver. The data are presented as the mean ± SEM (*n* = 8). Statistically significant differences are indicated; * *p* < 0.05 and ** *p* < 0.01.

To further investigate the mechanism of glucose metabolism under cold exposure, wortmannin was injected intraperitoneally into mice. The level of liver AKT, GSK3β, and PFKFB2 protein expression and phosphorylation were measured. As shown in Figure 8, wortmannin reduces the increase in p-AKT (Figure 8A; *p* < 0.05), p-GSK (Figure 8B; *p* < 0.05), and p-PFKFB2 (Figure 8D; *p* < 0.01) protein expression induced by cold exposure, and enhanced p-GS (Figure 8C; *p* < 0.01) protein expression.

### 2.9. CIRP Silencing Inhibits AKT Activity

**Figure 9:**
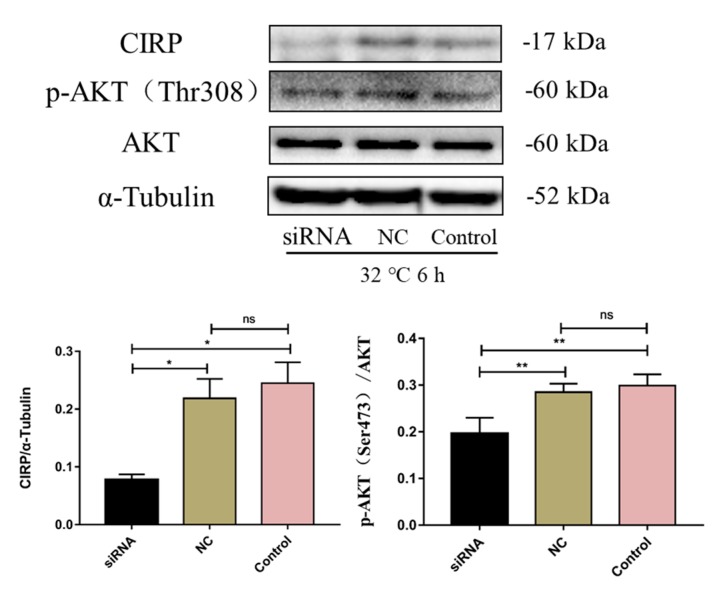
Effect of CIRP silencing on the level of protein kinase B (AKT) protein and phosphorylation status (Thr308) in C57BL/6 mouse livers. The data are presented as the mean ± SEM (*n* = 8). Statistically significant differences are indicated; * *p* < 0.05 and ** *p* < 0.01.

To verify the role of CIRP in the changes of glucose metabolism induced by acute cold exposure, AML-12 cells were transfected with CIRP siRNA. The level of CIRP protein expression and AKT phosphorylation were measured by Western Blot. As shown in Figure 9, CIRP silencing significantly reduces AKT phosphorylation levels.

## 3. Discussion

It has been shown that cold exposure causes variations in several physiological responses (e.g., resting energy expenditure and substrate metabolism) [28]. In addition, the liver acts as the primary metabolic organ involved in metabolic adaptive regulation, maintaining body glucose homeostasis, and plays an important role in the body’s energy metabolism. In the present study, we evaluated the level of blood glucose, insulin, and glucagon following cold exposure. The level of blood glucose was found to significantly decline following acute cold exposure. Moreover, glucose homeostasis is tightly regulated by insulin and glucagon. The level of glucagon continued to increase throughout the period of cold exposure; however, the level of insulin fluctuates during acute cold exposure. Glucagon is a major stimulator of gluconeogenesis and hepatic glycogenolysis and also promotes glycogen breakdown in the liver [29]. Insulin is also a major glucose-regulating hormone. Moreover, insulin and glucagon have been found to collaborate to control the rate of glucose utilization and production to maintain a euglycemic state [29]. In the present study, we found that the level of blood glucose was significantly reduced following 4 h of cold exposure. One possible reason is that all of the tissues except the liver increase their glucose uptake following cold exposure [30], and their energy expenditure increases up to three- to five-fold above the resting energy expenditure [28]. The regulation of insulin and glucagon is insufficient to maintain constant blood glucose. 

It has been well accepted that energy depletion leads to the suppression of protein synthesis and activation of endoplasmic reticulum (ER)-stress signaling [31]. Cold exposure increases glucose uptake and consumption in peripheral tissues [32], and the release of hepatic glucose increases to meet the body’s energy requirements. Excessive energy requirements may lead to energy depletion and apoptosis in liver cells. In addition, hepatocyte apoptosis is an important component of liver tissue failure and liver damage [33]. It has been suggested that cells with impaired mitochondrial energy metabolism reach an energy threshold, which triggers apoptosis [34]. Studies have shown that liver glycogen is dramatically reduced by exposure to −15 °C for 4 h [5], and 5 min of −20 °C cold exposure can induce liver edema and histopathological damage in rats [1]. In this study, we also found that the liver glycogen content decreased sharply after acute cold exposure, whereas cleaved caspase 3 did not change.

AKT is a central signaling molecule involved in a variety of intracellular signal transduction pathways and plays diverse cellular roles, including cellular growth, proliferation, metabolism, survival, angiogenesis, and migration [35]. Previous studies have reported that ER stress-induced hepatocyte death is dependent on AKT inhibition [36], and activation of the AKT pathway participates in the protective effects of liver injury [37,38]. In this context, we examined the activity of the AKT pathway following cold exposure.

The results of this study indicate that the phosphorylation of AKT and GSK3β was significantly higher in the livers of mice following cold exposure, the phosphorylation status of GS was reduced. GSK3β was phosphorylated and deactivated by AKT, which led to the activation of GS via dephosphorylation [29]. Thus, the major signaling pathways for glycogen synthesis in the liver were activated. This result may be due to the fact that cold exposure increases the consumption of hepatic glycogen, resulting in decreased glycogen content and an increased demand for glycogen synthesis. These findings agree with previous observations that acute cold exposure increases key enzymes required for liver glycogen synthesis [5]. 

PFKFB-2 is a bifunctional enzyme that catalyzes the production and destruction of fructose-2,6-bisphosphate. Fructose-2,6-bisphosphate is the physiologic allosteric activator of PFK-1, the rate-limiting enzyme of glycolysis [39], which catalyzes fructose-6-phosphate to fructose-1, 6-bisphosphate. Fructose-1,6-diphosphate is a metabolic intermediate that promotes cell metabolism, stimulates the process of glycolysis, provides sufficient adenosine triphosphate levels, and sustains intracellular calcium levels [40,41]. In summary, PFKFB-2 plays an integral role in the regulation of glycolysis. We noted that PFKFB-2 was activated by phosphorylation following cold exposure, which indicates that glycolysis activity is enhanced after cold exposure. Concomitantly, we have also found that fructose-1,6-diphosphate and pyruvate in the liver were increased during 0–2 h of cold exposure followed by a sustained decrease. FDP is the major intermediate metabolite of glycolysis and PA is the end product of glycolysis [40]. Thus, elevated FDP and pyruvate levels following cold exposure are indicative of increased levels of glycolysis, and the decreased levels after 2 h of cold exposure may be due to excessive energy requirements and substrate deficiencies.

Bcl-2 and Bax are important members of the Bcl-2 family, which control the intracellular signals that promote apoptosis and anti-apoptosis, and plays an important role in regulating endogenous apoptosis [42]. The anti-apoptotic protein, Bcl-2, and pro-apoptotic protein, Bax, balance cell survival and apoptosis by controlling mitochondrial outer membrane permeabilization [43,44]. Hypothermic conditions have been reported to significantly up-regulate the expression of the anti-apoptotic protein, Bcl-2, in endothelial cells [45]. In this study, our findings show that acute cold stress had no effect on Bax, but induced increased levels of Bcl-2. Moreover, the Bcl-2/Bax ratio increased in the liver of cold-exposed mice. These observations indicate that cold exposure activates the AKT-signaling pathway, which in turn affects the expression of glucose metabolism-related genes and anti-apoptotic genes in the livers of mice.

As a stress-responsive protein, the expressions of CIRP will up-regulate and respond to environmental signals under certain conditions of stress, allowing cells or organisms to quickly adapt to new environments. CIRP is associated with several signal transduction pathways required for cellular function, which are associated with both apoptosis and proliferation [46,47]. It has been reported that CIRP activates both the MAPK and NF-κB signaling pathways [48,49] and induces TRX expression [50]. In addition, CIRP overexpression was associated with reduced levels of p53, thereby downregulating proapoptotic genes and upregulating anti-apoptotic genes [51]. When the body was in stress states, CRIP expression was increased immediately to protect the homeostasis. It had been reported, CRIP would medicate by AKT signaling pathway, CIRP increased the level of p-AKT and activated AKT signal transduction pathway to reduce the level of apoptosis [52].

In our previous studies, CIRP inhibited neuronal apoptosis by blocking the formation of oxygen free radicals under low temperature conditions [26]. In the present study, the expression of CIRP in the livers of mice was up-regulated following acute cold exposure. To further verify the role of CIRP in acute cold exposure-induced changes in glucose metabolism, AML-12 cells were transfected with CIRP siRNA. After CIRP silencing, p-AKT expression was decreased and the AKT signaling pathway was inhibited. The results in our study were consistent with previous research.

In order to confirm the results between CRIP and AKT, we injected the inhibiter of AKT in mice and measured a series of indexes. The results demonstrated that the expression of p-AKT was decreased and activated level was inhibited, and the signaling pathway of apoptosis was increased remarkably compared with the control group. The phenomenon above confirmed our results in vitro. It was proved that AKT plays a key role in the process of cold stress and the CRIP could influence the activity of AKT. Finally, can conclude that CIRP regulates glucose metabolism by enhancing AKT-signaling pathway activity.

**Figure 10:**
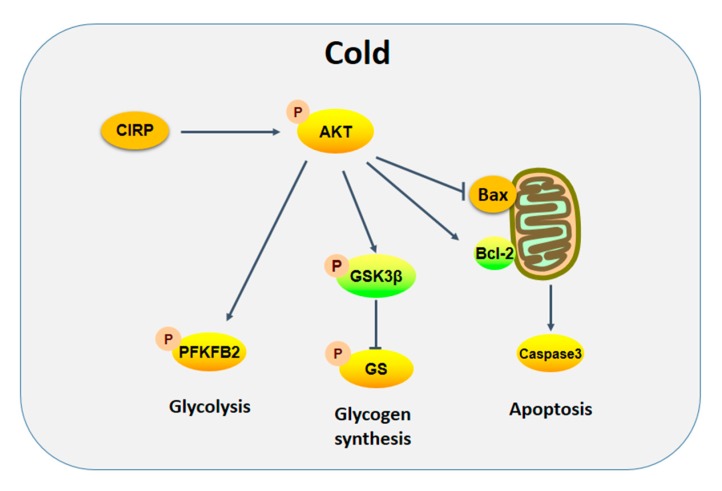
A proposed model for the molecular mechanisms of acute cold stress in the liver. Arrows express promotion, T-bar represent inhibition.

In conclusion, the findings of the present study demonstrate that acute cold exposure increases the expression of CIRP protein, which may regulate the hepatic glucose metabolism and maintain a hepatocyte energy balance through the AKT-signaling pathway under conditions of cold exposure, thereby slowing liver cell apoptosis caused by cold exposure. A proposed role of CIRP in acute cold stress in the liver is demonstrated in Figure 10.

## 4. Materials and Methods

### 4.1. Animals

Male specific pathogen-free C57BL/6 mice (seven weeks old; 30–32 g) were purchased from the Experimental Animal Center of PLA Academy of Military Medical Sciences (Shenyang, China). All mice were raised in an artificial climate chamber with controlled light and temperature. The mice were supplied with a restricted diet (fed from 08:00 p.m. to 08:00 a.m.) and had free access to water under a 12 h light–dark cycle at a constant temperature (28 ± 1 °C) and humidity (60 ± 10%). Prior to the experiment, mice were acclimatized to the laboratory conditions for a week. The study was approved by the Institutional Animal Care and Use Committee of Heilongjiang Bayi Agricultural University (Daqing, China, approved date: 5 February 2019). 

### 4.2. Cold Exposure Experiment, Core Body Temperature Measurement and Sample Collection

**Figure 11:**
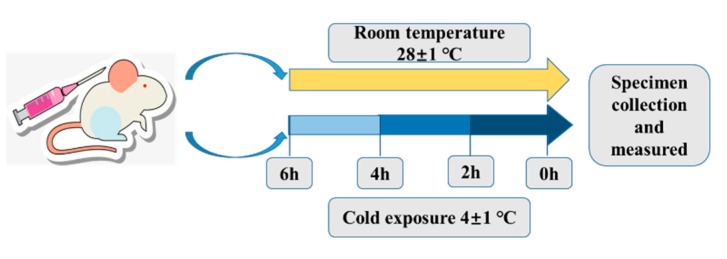
Study Protocol. Mice in the room temperature (RT) control group were left undisturbed, whereas cold-exposed mice were exposed to 4 °C.

The conditions of cold exposure have been previously described [53]. At the beginning of the experiment, mice were randomly distributed into six groups (eight mice per group): (A) control group, (B) 2 h cold exposure group, (C) 4 h cold exposure group, (D) 6 h cold exposure group, (E) wortmannin injection group, and (F) normal saline injection group. Wortmannin (0.5 mg/kg) was dissolved in dimethylsulfoxide (DMSO) and injected intraperitoneally into each mouse of the wortmannin injection group 1 h before cold exposure. As a control, NS containing the same concentration of DMSO was injected into the normal saline injection group mice. The study protocol is presented in Figure 11. The control group was maintained at 28 ± 1 °C. Groups B–F were exposed to the cold (4 ± 1 °C) in the artificial climate chamber for 2 h, 4 h, and 6 h, respectively. After terminating cold exposure, core body temperature was measured, then the livers and serum were harvested from mice, and the mice were euthanized. A part of the mouse livers was immediately fixed with 4% paraformaldehyde, while the remaining sections were immediately frozen in liquid nitrogen and placed in RNase- and DNase-free 1.5 mL Eppendorf tubes, and preserved at −80 °C until use. To avoid the influence of diurnal cycling, all samples were collected at approximately the same time each day.

### 4.3. Cell Culture and Treatment

AML-12 cell line was supplied by National Key Basic Animal Medicine Laboratory of Heilongjiang Bayi Agricultural University (Daqing, China). The cells were cultured at 37 °C in 5% CO_2_ in DMEM medium (#SH30022.01, Hyclone, Logan, UT, USA) and supplemented with 10% fetal bovine serum (#FB15015, CLARK Bioscience, Richmond, VA, USA). 

### 4.4. siRNA Transfections

CIRP small interfering RNA (siCIRP) was obtained from Shanghai Sagon Biotech (Shanghai, China), with the sequences: Sense, 5′-GGUCCUACAGAGACAGCUATT-3, and antisense, 5′-AGACUUCCCAUUCAUAGCCTT-3′. AML-12 cells were transfected with 50nM siRNA, using 10 µL Lipofectamine 2000 (Invitrogen; Thermo Fisher Scientific, Waltham, MA, USA), and were plated simultaneously onto 6-well plates. AML-12 cells were cultured at 32 °C for 6 h after transfection 72 h.

### 4.5. Measurement of Biochemical Parameters 

The level of serum fasting glucose was measured using commercial cards (IDEXX laboratories. Westbrook, ME, USA). Serum insulin and glucagon levels were measured using a commercial ELISA kit (Cloud-Clone Corp, Katy, TX, USA). Fructose-1,6-diphosphate (FDP) and pyruvic acid (PA) in the liver were measured using a commercial kit (Solarbio, Beijing, China).

### 4.6. PAS Staining

Formalin-fixed livers were embedded in paraffin after routine clearing and dehydration, and sections (5 µm thick) were prepared from paraffin-embedded tissue blocks using a rotary microtome. PAS staining was performed using a Periodic Acid Schiff/PAS Stain Kit (Solarbio, China) according to the manufacturer’s instructions. Briefly, after the sections were deparaffinized and rehydrated, they were incubated in 3% H_2_O_2_ for 15 min at room temperature to block endogenous peroxidase activity. The sections were each stained with Periodic Acid Schiff (PAS) reagent to examine the glycogen content of liver tissues.

### 4.7. Western Blot Analysis

Liver tissue specimens were homogenized with a ground glass tissue grinder in RIPA lysis buffer (#P0013B, Beyotime, Shanghai, China) containing protease and phosphatase inhibitors (PMSF) (#ST506, Beyotime, Shanghai, China), then lysed for 30 min on ice to prepare protein extracts. The extracts were sonicated and centrifuged at 16,060 rcf for 15 min at 4 °C. The concentration of the protein in each sample was determined with BCA protein assay reagent (#P0010, Beyotime, Shanghai, China), reconstituted in lysis buffer to a uniform protein concentration, then boiled for 10 min in the presence of a loading buffer. An equal amount of protein was separated by sodium dodecyl sulfate-polyacrylamide gel electrophoresis, and thereafter, electrotransferred to a PVDF membrane (0.45 μm, Millipore, Darmstadt, Germany). The membranes were blocked in 5% nonfat milk in Tris-buffered saline containing 0.05% Tween-20 (TBST) for 1 h at room temperature, then incubated with the primary antibodies overnight at 4 °C. Membranes were removed into TBST and washed three times for 10 min, after which they were incubated with the secondary antibody for 2 h at room temperature. The membranes were washed three times as previously described. After washing, the membranes were imaged using an ECL kit and ChemiDoc XRS (Bio-Rad, Hercules, CA, USA) and analyzed with ImageJ software (http://imagej.nih.gov/ij/, accessed on 22 March 2019). Alpha-tubulin was used as the reference protein.

CIRBP (1:17000, #ab166779) and GSK3β (1:8000, #ab32391) antibodies were purchased from Abcam (Amyjet Scientific Inc., Upper Heyford, UK). AKT (1:1000, #90272), phospho-AKT (Ser473) (1:1000, #12694), PFKFB2 (1:1000, #13045), phospho-PFKFB2 (Ser483) (1:1000, #13064), phospho-GSK3β (Ser9) (1:1000, #9323), GS (1:1000, #3886), and phospho-GS (Ser641) (1:1000, #3891) antibodies were purchased from Cell Signaling Technology (Danvers, MA, USA). Bax (1:2000, #60267-1-lg), Bcl-2 (1:2000, #60178-1-lg), and caspase 3 (1:500, #66470-2) antibodies were purchased from Proteintech (Rosemont, IL, USA). Secondary antibodies were labeled with horseradish peroxidase goat anti-mouse IgG (1:20000, # SA00001-1, Proteintech, Rosemont, IL, USA), goat anti-rabbit IgG (1:20000, #SA00001-2, Proteintech, Rosemont, IL, USA), and goat anti-mouse IgG, IgM, and IgA (1:10000, ThermoFisher, #A-10668).

### 4.8. Statistical Analysis

All data are expressed as the means ± standard error of the mean (SEM). Statistical analysis of the data was performed using GraphPad Prism software (La Jolla, CA, USA). Significant differences were evaluated by a one-way analysis of variance (ANOVA). For all analyses, post hoc comparisons were made using Fisher’s Least-Significant Difference (LSD) post hoc test. A *p* value < 0.05 was considered statistically significant.
